# Breakfast Consumption and Quality of Macro- and Micronutrient Intake in Indonesia: A Study from the Indonesian Food Barometer

**DOI:** 10.3390/nu15173792

**Published:** 2023-08-30

**Authors:** Helda Khusun, Roselynne Anggraini, Judhiastuty Februhartanty, Elise Mognard, Khalida Fauzia, Nursyifa Rahma Maulida, Ony Linda, Jean-Pierre Poulain

**Affiliations:** 1 Faculty of Health Sciences, University of Muhammadiyah Prof. Dr. HAMKA, Jakarta 12130, Indonesia; nursyifa.maulida@uhamka.ac.id (N.R.M.); ony_linda@uhamka.ac.id (O.L.); 2 Southeast Asian Ministers of Education Organization Regional Centre for Food and Nutrition (SEAMEO RECFON)—Pusat Kajian Gizi Regional (PKGR), Universitas Indonesia, Jakarta 13120, Indonesia; lynne.prigel@gmail.com (R.A.); jfebruhartanty@seameo-recfon.org (J.F.); khalidafauzia@seameo-recfon.org (K.F.); 3 Nutrition Department, Faculty of Medicine, Universitas Indonesia—Dr. Cipto Mangunkusumo General Hospital, Jakarta 10430, Indonesia; 4 Chair “Food Studies: Food, Cultures & Health”, Taylor’s University (Malaysia), Subang Jaya 47500, Malaysia; eliseline.mognard@taylors.edu.my (E.M.);; 5 Centre for Asian Modernisation Studies (CAMS), Taylor’s University (Malaysia), Subang Jaya 47500, Malaysia; 6 Faculty of Social Sciences and Leisure Management, Taylor’s University (Malaysia), Subang Jaya 47500, Malaysia; 7 Centre d’Études et de Recherche: Travail, Organisation, Pouvoir (CERTOP) UMR CNRS 5044, Université de Toulouse (France), 31000 Toulouse, France

**Keywords:** breakfast consumption, diet quality, Indonesia, nutrient intake, nutrient-rich food index 9.3

## Abstract

Breakfast is an important meal that has been shown to have a positive effect on health. The current study aimed to assess the patterns of breakfast consumption among adult Indonesians and to estimate the contribution of breakfast to their nutrient intake and dietary quality. The study used 24-h recall data from the 2018 Indonesian Food Barometer study to assess breakfast intake among 1333 adults aged 18 and above from six provinces in Indonesia. Diet quality was measured using the Nutrient Rich Food index (NRF) 9.3, and the nutritional profile of breakfast was compared across tertiles of NRF 9.3. In total, 5.2% of adults in Indonesia skipped breakfast. Breakfast contributed 26% to daily energy intakes and 22–28% to intakes of all reported nutrients, except for total sugar (12%), vitamin C (8%) and vitamin D (7%). With respect to daily requirements, breakfast contributed approximately 20% to energy, protein, fat and sodium requirements, 26% to saturated fat but <15% to the requirements for most micronutrients and only 5% for fiber. Among breakfast consumers, a higher NRF score was associated with higher daily intakes of protein, dietary fiber and micronutrients and lower intakes of sodium from breakfast. This study suggests that a balanced breakfast in Indonesia should aim to lower fat and saturated fat intake while increasing fiber, potassium, calcium and vitamin C and D intake. These findings could inform the development of nutrient-based guidelines for breakfast consumption in Indonesia.

## 1. Introduction

Indonesia is currently facing the triple burden of malnutrition, with undernutrition and over nutrition existing side by side and coupled with micronutrient deficiency [1]. The last National Total Diet Study in 2014 showed that consumption patterns of Indonesians are still dominated by cereal and plant protein sources with very low adherence to fruit and vegetable recommendations, while the consumption of sugar, salt and fat is already approaching maximum recommended levels [2,3]. Several studies among women and men have shown that the overall diet quality among Indonesians using different diet quality measures is considered low [4,5,6] or as needing improvement [7] with respect to meeting the Indonesian dietary guidelines [8].

The Indonesian dietary guidelines enacted in 2015 have 10 nutrition messages, 1 of which is a recommendation to eat breakfast every day [8]. In the guidelines, breakfast is defined as eating and drinking activities from waking-up in the morning to 09:00 a.m., and it should meet about 15 to 30% of the Recommended Dietary Allowance (RDA) to promote healthy, active and productive living [8]. Past evidence has revealed the importance of breakfast and its positive effect on dietary quality and health condition. Studies in other countries revealed that breakfast irregularity or skipping was associated with lower nutrient intake and diet quality [9,10,11,12,13,14], higher risk of obesity [14,15] and poorer school performance [16]. Moreover, a meta-analysis of observational studies among adults showed that skipping breakfast was related to an increased risk of type 2 diabetes mellitus [17]. 

Indonesian national data on breakfast consumption patterns is lacking, especially among adults. Several regional studies among children and adolescents reported breakfast skipping rates between 41 and 58% among this age group [18,19] and that when consumed, the breakfast quantity and quality was poor [19,20]. The contribution of breakfast toward carbohydrate and fat requirements was high but low for fiber and micronutrients [20]. Furthermore, lower breakfast quality was associated with lower socioeconomic status [20,21].

The International Breakfast Research Initiative (IBRI) was set up in 2017 to examine breakfast consumption patterns in different parts of the world, with the aim of developing nutrient recommendations for breakfast on a regional basis. The aim of the current paper is to assess breakfast consumption patterns and quality among Indonesian adults in the context of the IBRI. These data will serve to provide a proposal on breakfast recommendations to policy makers based on the observed practices. 

## 2. Materials and Methods

### 2.1. Data Source and Study Sample

This study was a secondary data analysis of the 2018 Indonesian Food Barometer (IFB) study conducted by SEAMEO RECFON. Data acquisition was carried out by submitting a formal request to SEAMEO RECFON for the use of their data. The IFB was a cross-sectional survey conducted in six (6) provinces of Indonesia, where 48% of the Indonesian population resides (West Sumatra, Jakarta, West Java, East Java, Bali and South Sulawesi) [22]. The survey consisted of 1665 adults aged more than 18 years who were randomly selected using multistage cluster random sampling. The sample selection was designed to represent urban and rural population in the six (6) provinces included, with oversampling of urban areas to allow for comparison if needed. Of the 1665 subjects, 332 participants were excluded based on under- and overestimation of energy intake using Goldberg Criteria [23]. Thus, current analysis used 1333 subjects. Training for interviewers was conducted to ensure common implementation of fieldwork, data collection and input. Data were collected from December 2017 to March 2018. The protocol was approved by the Human Ethics Committee of Faculty of Medicine Universitas Indonesia (reference 927/UN2.F1/ETIK/V/2017). Written informed consent was obtained from all subjects. Detailed information on this study’s design, protocol and methodology has been previously published [24,25,26,27]. 

### 2.2. Dietary Assessment

Dietary intake data were collected by face-to-face interview using a structured questionnaire to collect information on all food and beverages consumed over a 24-h period. The multiple-pass method was used to record all the foods and drinks consumed the previous day of the survey, from waking up until going to bed again at night. Food photographs were used to aid the estimation of portion sizes [28]. For each food occasion on the day of the interview, participants were asked to name the eating occasions themselves. Data collected represent all days in a week, including the weekend, to have a population picture of the food consumption. 

Energy and nutrient intakes were calculated using a customized version of the NutriSurvey for Windows 2007 software. The Indonesian Food Composition Tables (FCT 2017) were used to estimate almost all of the nutrients. We also adopted the Philippines FCT 2019 to complete the total sugar, saturated fatty acid, magnesium, vitamin E and vitamin D. For mixed dishes and food not available in the Indonesian FCT, a recipe approach was used to estimate nutrient content based on raw ingredients from the Indonesian FCT. Two recipes from cookbooks and/or from the Internet were reviewed, and the research team decided on the more plausible recipes to be used. The recipes were then used to calculate the nutrient content of the food. The calculation of nutritional content was determined by the quantity of ingredients required to create a 100 g edible portion of the dish in its serving-ready state. This calculation involves the nutritional information per 100 g of the individual ingredients and takes into account the yield factors and nutrient retention factors, following the guidelines for recipe information and calculation of nutrient composition of prepared foods (dishes) by Bognar and Piekarski [29]. 

Quality control was conducted at several stages. During the fieldwork, a supervisor monitored the result of interviews and completeness of the 24-h recall form on a daily basis. The enumerator returned to the subject if the recall form was found to be incomplete. Quality control of the reported nutrient intakes was checked for outliers and implausible energy intake. Participants with implausible energy intake based on Goldberg criteria were excluded [23].

### 2.3. Breakfast Definition

As previously reported, defining the breakfast meal in dietary surveys can be challenging [30,31]. The Indonesian dietary guidelines define breakfast as food consumed before 9:00 a.m. that provide 15–30% of daily nutrient needs [8]. For the current study, breakfast was defined as the “first meaningful” intake of food and/or drinks consumed before 10:00 a.m., reported from the single 24-h food recall. This definition aims to be in line with previous research studies of the International Breakfast Initiative [30], the definition from the Indonesian Dietary Guidelines [8] and the analysis of the local organization of the food day. The term “meaningful” criteria used in this study was an energy intake of more than 50 Kcal [32,33,34]. If there was more than one food occasion fulfilling the breakfast definition, the selection of the food occasion to be defined as breakfast was the food occasion defined as breakfast or morning snack by the respondents or the food occasion having an energy intake nearest to 15% of the daily energy recommendation. Breakfast skippers were defined as those who did not report any intake before 10:00 a.m. or had intake before 10:00 a.m. with an energy content of fewer than 50 Kcal.

### 2.4. Dietary Quality by NRF 9.3

The Nutrient-Rich Food Index (NRF) 9.3 is a validated nutrient profiling method to measure the quality of overall diet [35]. The NRF index calculates nutrient density based on 12 nutrients and total energy intake. These nutrients were divided into 9 qualifying nutrients (NRs) and 3 disqualifying nutrients (LIM). Nutrient references values for 11 nutrients were based on the Nutrition Label’s Reference for Indonesian 2016 [36], whereas the total sugar followed the recommendation stated in the Ministry of Health Regulation no 30, year 2013 [37].

The qualifying nutrients and reference daily values (DVs) are as follows: protein (60 g), magnesium (350 mg), dietary fiber (30 g), vitamin A (600 RAE), vitamin C (90 mg), vitamin D (15 mcg), potassium (4700 mg), calcium (1100 mg) and iron (22 mg). The disqualifying nutrients and maximum recommended values (MRVs) are sodium (1500 mg), saturated fat (20 g) and total sugar (50 g). The daily energy intake for adults was 2150 Kcal. 

The NRF 9.3 was calculated as follows:

(1) $$NRF\;9.3=\left(NR-LIM\right)\times 100$$

(2) NR=∑i=19intakeienergy × 2150DVi

(3) LIM=∑i=13intakeienergy × 2150MRVi−1

where intake *i* is the intake of each nutrient *i*.

Following the calculation instruction, NRs were truncated at 1, so that an excessively high intake of one nutrient could not compensate for the dietary inadequacy of another. Also, in LIM, only the share in excess of the recommended amount was considered. 

### 2.5. Statistical Analysis

To ensure representativeness of the Indonesian population, the sample was weighted by population density, urban–rural location, age and sex composition, taken from the 2010 Indonesian Census. 

Statistical analysis was conducted using SPSS v20 software (IMB, New York, NY, USA). Data were presented as both continuous and categorical. For continuous data, normality was assessed by the Kolmogorov–Smirnov statistic. Due to the skewness of the data, nutrient intakes were presented in median and 25th–75th percentile. 

The relationship between socio-descriptive categorical variables and breakfast consumption was examined using chi-square test. The differences in quantitative variables (such as nutrient and energy intakes) were tested using Analysis of Covariance (ANCOVA) for controlling for confounders, including energy intake at breakfast, gender, age, marital status, metropolization and occupation, adjusted where appropriate. An LSD post hoc test was used to examine multicomparisons between groups. The significance level was set at *p* < 0.05.

## 3. Results

The sociodemographic details of the study population are shown in Supplementary Table S1. The proportion of subjects residing in urban areas was 65.6%. The study population’s age ranged from 18 to 88 years, with the majority of subjects aged 30–59 years (57.7%). The study participants had comparable proportions between sexes, and 69.3% of them were married. Around 45% of the subjects had low education (elementary and junior high school with less than or equal to 9 years of education). Muslims made up the majority of the respondents (92.7%).

Following the breakfast definition, 5.2% of the respondents were categorized as breakfast skippers. Breakfast skipping was significantly higher in the urban population (6.5%), among adult 18-29 years (7.3%), among nonmarried individuals (8.9%) and those who were underweight (10%) (Table S1).

**Figure 1:**
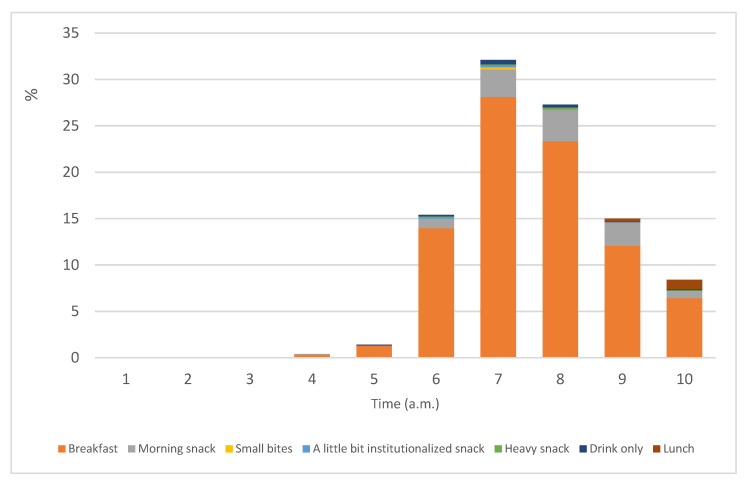
Time and type of morning intake among breakfast consumers (n = 1263).

Figure 1 shows the various names assigned by the breakfast consumers (n = 1263) to their food intake before 10:00 a.m. The majority of the respondents named the intake consumed prior to 10:00 a.m. as breakfast. Snacks, particularly morning snacks and heavy snacks, were other words used to describe morning consumption. Most respondents consumed breakfast at around 7:00 a.m. (32.1%), followed by 8:00 a.m. (27.3%). Few had breakfast in the early hours, including those who had meals before sunrise (*sahur*)during fasting for Muslims. 

### 3.1. Contribution of Breaksfast to Daily Energy and Nutrient

Table 1 shows that median daily energy intake was 1626 Kcal, while median intake of energy from breakfast was 396 Kcal. The median daily energy intake was significantly higher among breakfast consumers than breakfast skippers (1639.4 (25th–75th percentile: 1318.7–2120.3) vs 1429.8 (1237.2–1791.6)). The proportions of energy from carbohydrate, sugar, protein, fat and saturated fat for the daily energy intake were 51.9%, 6.5%, 13.8%, 33.6% and 12.8%, respectively. Meanwhile, the contributors of energy during breakfast were 55.2% from carbohydrate, 1.5% from sugar, 12.7% from protein, 31.2% from fat and 12.2% from saturated fat.

**Figure 2:**
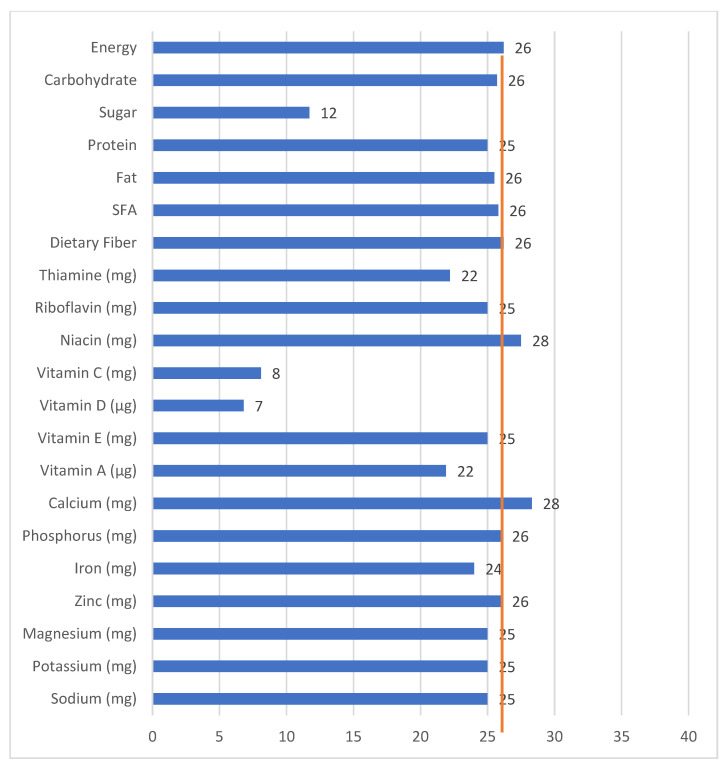
Contribution (%) of macro- and micronutrient intake during breakfast to total daily intake among breakfast consumers (n = 1263). Note: The vertical line is the median percent of daily energy intake consumed at breakfast.

Among breakfast consumers, the energy and macronutrient intakes at breakfast contributed to around 25% of daily macronutrient intake, except for sugar intake, which contributed 11.7% (Figure 2). All micronutrient intake during breakfast, except for vitamin C and D, contributed to around 21–28% of daily intake. Vitamin C and vitamin D intake during breakfast was very low, contributing to only 8.1% and 6.8% of daily intake, respectively.

### 3.2. Contribution of Breaksfast to Recommended Daily Allowance (RDA)

**Figure 3:**
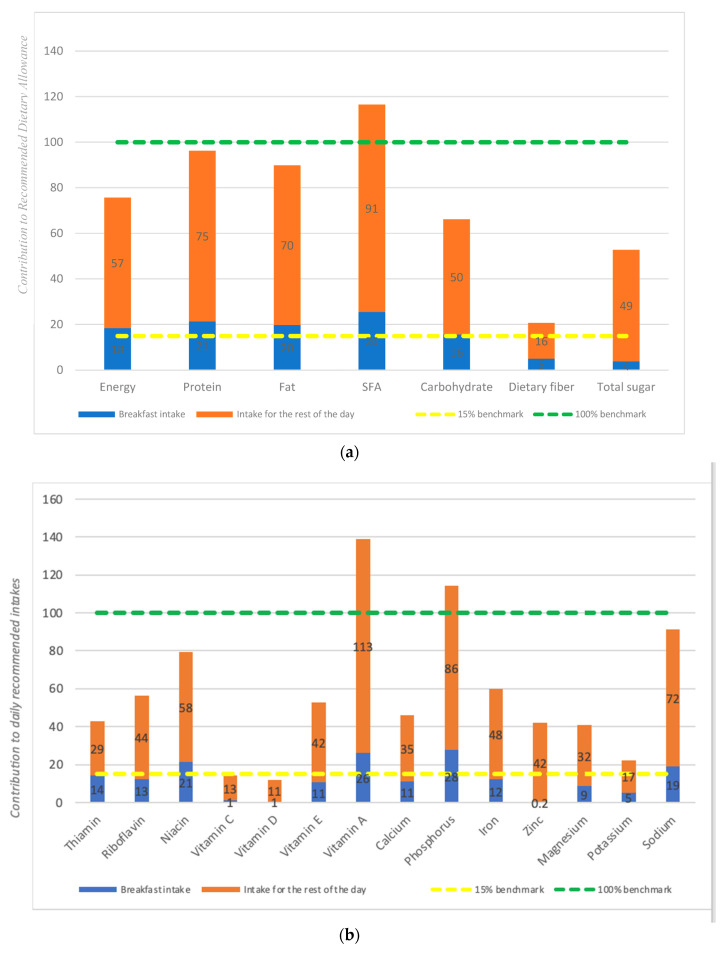
(**a**) Percent contribution of median intakes of macronutreint at breakfast (blue box) and for the total day (blue and orange boxes) to the daily recommendation allowance (represented by the green line). (**b**) Percent contribution of median intakes of micronutrient at breakfast (blue box) and for the total day (blue and orange boxes) to the daily recommendation allowance (represented by the green line). Note: Yellow dashed line is set at the 15% benchmark, which is the minimum nutrient contribution expected from breakfast according to the Indonesian Dietary Guidelines.

Figure 3a,b shows the adequacy of macro- and micronutrient intake against the RDA (marked by green dashed line 100% benchmark) and how much breakfast intake contributed to the RDA. The yellow dashed line shows the 15% benchmark, which was the minimal nutrient contribution expected from breakfast according to the Indonesian Dietary Guidelines [8]. The daily adequacies of median energy and carbohydrate intake were 78% and 66%, respectively, and breakfast contributed 18% and 16% toward the RDAs, respectively. The daily adequacies of protein and total fat reached 98% and 90%, respectively, and the contribution of breakfast intake was around 20%. Daily intakes of saturated fatty acids exceeded the RDA, with breakfast contributing 26%. Daily intakes of dietary fiber reached only 21% of the RDA, with breakfast contributing to only 5%. Total sugar intake from this study was 53% of the recommended allowance of less than 50 g per day, and breakfast contributed only to 4% of this recommendation. Among all micronutrients in Figure 3b, only vitamin A and phosphorus intakes reached the RDA, while sodium intake was close to the maximum recommendation. The lowest daily adequacy of micronutrient intake was found for vitamin D (12%), vitamin C (14%) and potassium (22%). Breakfast contributed to more than 20% of the RDA for niacin, vitamin A and phosphorus, while all other micronutrient intakes during breakfast were less than 15% of the RDA.

### 3.3. Breakfast Intake and Diet Quality

Overall, the median NRF score of the study population was 383.5 (25th–75th percentile: 295.2–471.0). There were no statistically significant differences in diet quality as measured by NRF score between breakfast skippers and breakfast consumers (Supplementary Table S2). Furthermore, the NRF score was significantly lower among the younger population, females, those living in urban areas and single individuals and significantly higher among those having lower education, in the lowest wealth index and among the obese population (Supplementary Table S2). 

Table 2 describes the nutrient intake and food group consumption during breakfast across different levels of diet quality score as measured by NRF 9.3. Table 2 shows that subjects at the highest tertile of the NRF 9.3 score had significantly higher intakes of protein, dietary fiber, B vitamins, vitamin A, vitamin C, potassium, calcium, iron and magnesium and significantly lower sodium intakes from breakfast. Meanwhile, subjects at the lowest tertile of the NRF 9.3 score had significantly higher intakes of saturated fat. The intakes of total energy, fat, carbohydrate, vitamin D, vitamin E and zinc were not significantly different across the NRF 9.3 tertiles. 

Further analysis into the food groups (Table 3) showed that the most commonly reported foods consumed at breakfast were cereal grains (74.9%, mostly from rice), followed by vegetables (34.5%), legumes (33.3%), fish (17.8%), snacks (17.3%, e.g., traditional cakes and fritters), eggs (14.4%) and beverages (18.4%, e.g., tea and coffee). 

Subjects at the highest NRF tertile had a significantly higher median intake of vegetable and vegetable products, legumes and legume products and snack but lower median intakes of fish and shellfish and eggs and egg products. Meanwhile, subjects among the lowest NRF had a significantly higher median intake of beef and beef products. Moreover, there was a nonsignificant trend towards higher fruit and fruit juice consumption among the highest NRF tertile. 

## 4. Discussion

After sample weighting, the survey participants’ profiles represented the typical Indonesian population when compared with the 2020 Indonesian census (17). The prevalence of breakfast skippers among Indonesian adults in this study is 5.2%, which is somewhat similar with other parts of the world [32,38,39,40,41] but lower than that of Canadian adults [42]. The low prevalence of breakfast skippers signifies the importance of the meal to the Indonesian adult population. However, a much higher prevalence of breakfast skipping has been reported among Indonesian children and adolescents [18,19], which could be due to differences in the methodology and definition of breakfast used but also could reflect different eating patterns among these age groups. 

The observed socio-demographic profile of breakfast skippers who were younger adults, single and living in urban areas confirms the findings of previous studies in Europe and North and South America [32,40,41,42,43]. Food consumption habits change more rapidly among these groups, and these groups were also reported as those at the highest rates of nutrition transition [44].

### 4.1. Adequacy of Intake and Nutrient Contribution from Breakfast

The overall energy intake in this study was 1627 Kcal, which is similar to the result of a previous national survey [3] and accounts for 77% of the energy of the 2000 Kcal of the Indonesian RDA. Similarly, a meta-analysis of dietary surveys of pregnant women in both Indonesia and Malaysia showed that the energy intakes among Indonesian women averaged just 85% of the Indonesian RDA [45]. The extent of energy under-reporting in the present study using the Goldberg cut-off points [23] was found to be 25%, which compares favorably with similar data from the UK (28%) [46] and the US (25%) [47]. The adult female population in this study has an average weight and height of 58.4 ± 11.7 kg and 151.5 ± 5.8 cm, while for males it is 61.6 ± 12.0 kg and 163.5 ± 6.4, respectively. These weight and height averages are much lower than US adults [48] and, as such, the lower observed energy intake of 1624 Kcal is not surprising.

The energy intake of breakfast among adult Indonesians (396 Kcal) corresponded to 26% of daily energy intake. The energy contribution of breakfast to daily intake is similar to the results in Malaysia (26%) [39] and the Philippines (30%) [38] but higher compared with America (21.6–23%) and Europe (16–24%) [32,40,41,42,43]. The contributions of other macro- and micronutrients are similarly at the level of 21–25%, except for vitamin C (8,%), vitamin D (7%) and total sugar (12%). The low vitamin C intake is likely due to the low percent of consumers of vegetables (35%) and fruit at breakfast (3%).

When compared with the RDA, the current contribution of energy from breakfast (18%) was within the range of 15–30% adequacy, as stated in the Indonesian Dietary Guidelines [8]. The contribution of breakfast toward the RDA for other nutrients was not homogenous. However, by the definition used in this study, breakfast contributed to a total of 21–26% of daily macronutrient intake and 16–26% of the RDA, except for dietary fiber. Nevertheless, micronutrient adequacy regarding the RDA is low for most micronutrients, except for vitamin A and phosphorus. This indicates that most Indonesians consume high-energy breakfasts with low nutrient density. This result is in line with findings on low breakfast quality among children [21,49,50].

Previous studies in Indonesia, which are limited, showed the adequacy of vitamin C and vitamin A among different population groups was varied [51,52,53,54]. Different populations, age groups and the context of the study may lead to different results. Moreover, the use of single 24-h result leads to variability in vitamin intake assessment. A study among pregnant women in Indonesia showed that six (6) replicate recalls were needed for vitamin A and vitamin C intake assessment to be reliably estimated [55]. However, we believe that vitamin C consumption in the study population is low. The food group consumption showed that intakes of vegetables and fruits at breakfast were very low (45 g) if compared with the recommendation of 400 g/day, which is similar to the previous study [56]. The results from Indonesian basic health research also show that more than 95% of Indonesians do not consume fruits and vegetables as recommended [3]. Further study is needed to elucidate the different results of vitamin intake profiles in Indonesia. As for vitamin A, an analysis of the Total Diet study showed that the main food source for vitamin A was eggs, palm oils, carrots, water spinach and poultry [53].

Of particular interest was the contribution of breakfast toward total sugar intake, which was considered very low (1.5%). The total intake of sugar itself (26. 4 g/d) was found to be low compared with the maximum allowable intake of 50 g/day in Indonesia, but it slightly exceeded the WHO conditional recommendation of 25 g per day [57]. This result was similar to a previous national survey in Indonesia [2,3] and a previous study in the same six provinces [58]. However, the result from this study was lower than a recent study in Jakarta, which found that total sugar intake already reached more than 48 g/day, with beverages as the main contributor of sugar intake. The current study covers a much broader area, covering provinces which have a large rural area, such as East Java and South Sulawesi. The results indicate that half of Indonesian already has a sugar intake above the WHO conditional recommendation, and the situation may be worse in urban areas.

On the other hand, saturated fatty acid intake is already very high, and breakfast’s contribution toward the RDA is already 26%, approaching the maximum 30% benchmark. This is in line with previous studies in Indonesia which showed a high contribution of fat toward energy intake [2,59,60], as well as its association with obesity [61,62]. These studies results indicate that reducing fat intake is important and reducing the intake of fat during breakfast could improve overall daily fat intake.

### 4.2. Breakfast Intake and Diet Quality

Overall diet quality analyzed in the study as measured by NRF score was low, which is in line with previous studies on diet quality in Indonesia [63,64,65]. In terms of breakfast energy and nutrient intakes, the present analyses also allowed to identify food choices and breakfast patterns at different levels of diet quality, as captured by the NRF 9.3 scores. A higher NRF 9.3 score was associated with increased consumption of healthier foods, such as vegetables, legumes, fruits and fruit juice. The study found that better diet quality was characterized by higher intake of essential nutrients and a more diverse range of food groups compared with the lower tertiles.

The Indonesian government has launched efforts to improve overall diet quality and reduce sugar, salt and fat intake. A community campaign has been launched through the “Gerakan masyarakat hidup sehat” (Community Healthy Life Movement), known as GERMAS, which promotes a balanced diet based on the Indonesian Dietary Guidelines. Moreover, efforts to regulate sugar, salt and fat in processed foods have been enhanced by the obligatory insertion of a nutrition fact panel and voluntary use of the Healthier Food Choice Logo since 2019 [66]. Nonetheless, the impact of these initiatives has not been properly evaluated and, to our knowledge, no specific attention has been given to the breakfast meal.

## 5. Conclusions

With the breakfast definition used in the study, 5.2% of respondents were categorized as breakfast skippers. Breakfast skipping was significantly higher in the urban population (6.5%), among adults aged 18–29 years (7.3%), among nonmarried individuals (8.9%) and those who were underweight (10%). Furthermore, breakfast quality was significantly lower among the younger population, females, those living in urban areas and single individuals. Breakfast contributed roughly 396 Kcal and 26% of the daily energy in the Indonesian diet, which is high in fat and saturated fat. Saturated fat, vitamin A and sodium intakes are particularly high at breakfast. In contrast, intakes of fiber, zinc, vitamins C and D, potassium, magnesium, iron and several B-vitamins are low at breakfast but also for the total day. Although breakfast consumption versus skipping was not directly associated with the NRF 9.3 score, this study shows that a higher level of NRF 9.3 score was associated with a better nutrient profile of breakfast, which may help with the creation of nutrient-based guidelines for a balanced breakfast in Indonesia tailored to overcome the current gap in nutrient adequacy. The nutrient-based guidelines for a balanced breakfast are expected to improve breakfast quality, which in turn can improve overall diet quality. Nonetheless, further research is needed to translate the nutrient-based guidelines for balanced breakfast into food-based guidelines, which may be more easily interpreted by consumers. Moreover, more intervention studies are needed to improve breakfast quality based on the current adequacy gap found in this study.

## Figures and Tables

**Table 1:** Median intakes of macro- and micronutrients at breakfast and for the total day among Indonesian adults.

	Breakfast Intake (n = 1263)	Daily Intake (n = 1333)
	Median (25th–75th)	Median (25th–75th)
Macronutrients		
Energy (Kcal)	395.8 (294.2–571.3)	1626.2 (1314.7–2085.1)
Carbohydrate (g)	50.9 (37.8–75.0)	215 (161.2–270)
Carbohydrate (E%)	55.2 (43.3–65.0)	51.9 (44.3–59.7)
Total sugar (g)	1.9 (0.5–8.3)	26.4 (11.7–43.4)
Total sugar (E%)	1.5 (0.5–9.4)	6.5 (2.9–10.4)
Protein (g)	12.8 (7.9–20.5)	57.7 (42.9–75.3)
Protein (E%)	12.7 (8.7–17.6)	13.8 (10.8–16.9)
Fat (g)	13.3 (7.6–23.4)	60.2 (43.0–8.6)
Fat (E%)	31.2 (21.4–41.1)	33.6 (26.8–41.0)
SFA ^1^ (g)	5.1 (2.4–10.2)	23.3 (15.7–33.6)
SFA ^1^ (E%)	12.2 (6.1–17.9)	12.8 (9.5–16.9)
Dietary fiber (g)	1.5 (0.5–3.0)	6.2 (3.5–10.1)
Micronutrients		
Thiamine (mg)	0.2 (0.1–0.3)	0.7 (0.5–1.1)
Riboflavin (mg)	0.2 (0.1–0.4)	1.0 (0.6–1.5)
Niacin (mg)	3.2 (1.3–5.6)	13.0 (8.8–18.7)
Vitamin C (mg)	1.0 (0–5.2)	12.8 (4.4–29.4)
Vitamin D (µg)	0.1 (0–0.9)	1.8 (0.5–3.6)
Vitamin E (mg)	1.6 (0.5–3.3)	7.9 (4.6–16.9)
Vitamin A (µg)	156.4 (59.2–356.8)	833.5 (515.3–1389.5)
Calcium (mg)	120.2 (65.0–222.0)	505.3 (345.4–721.4)
Phosphorus (mg)	195.6 (108.9–303.5)	800 (587.6–1062.3)
Iron (mg)	2.7 (1.7–5.0)	13.2 (9.7–17.8)
Zinc (mg)	1.3 (0.7–2.7)	6.2 (4.3–9.8)
Magnesium (mg)	30 (15.3–59.2)	143.4 (89.6–201.3)
Potassium (mg)	244.6 (93.3–467.6)	1048.0 (678.8–1871.0)
Sodium (mg)	285.7 (114.2–593.1)	1368.1 (836.0–2070.2)

^1^ SFA: saturated fatty acid.

**Table 2:** Median Breakfast Nutrient Intake by NRF Tertile.

	Total (n = 1263)	NRF Tertile 1 (n = 407)	NRF Tertile 2 (n = 440)	NRF Tertile 3 (n = 416)	*p*-Value	Adjusted *p*-Value ^2^
	Median (25th–75th)	Median (25th–75th)	Median (25th–75th)	Median (25th–75th)		
Energy (Kcal)	395.8 (294.2–571.3)	392.2 (299.3–568.2)	398.0 (279.8–543.4)	401.5 (31.9–595.8)	0.104	0.485
Macronutrients						
Carbohydrate (g)	50.9 (37.8–75.0)	51.1 (36.4–72.9)	48.9 (31.7–75.9)	54.1 (42.0–79.6)	0.007 *	0.900
Total sugar (g)	1.9 (0.5–8.3)	1.3 (0.3–8.3)	2.0 (0.5–10.5)	2.0 (0.7–6.5)	0.391	0.030 *
Protein (g)	12.8 (7.9–20.5)	12.2 (6.8–16.6)	12.6 (7.4–20.8)	15.2 (10.0–25.0)	<0.001 *	<0.001 *
Fat (g)	13.3 (7.6–23.4)	13.1 (7.7–22.0)	13.2 (7.8–22.2)	13.7 (7.2–24.9)	0.859	0.738
SFA ^1^ (g)	5.1 (2.4–10.2)	5.4 (2.5–12.9)	4.8 (2.7–9.2)	5.0 (2.3–9.1)	0.001 *	<0.001 *
Dietary fiber (g)	1.5 (0.5–3.0)	1.3 (0.4–2.7)	1.4 (0.5–2.7)	2.1 (0.6–4.3)	<0.001 *	<0.001 *
Micronutrient						
Thiamin (mg)	0.2 (0.1–0.3)	0.1 (0.1–0.2)	0.1 (0.1–0.3)	0.2 (0.1–0.3)	<0.001 *	0.015 *
Riboflavin (mg)	0.2 (0.1–0.4)	0.1 (0.1–0.3)	0.2 (0.1–0.4)	0.3 (0.2–0.5)	<0.001 *	<0.001 *
Niacin (mg)	3.2 (1.3–5.6)	2.8 (0.8–5.0)	3.1 (1.3–5.9)	4.3 (2.6–6.8)	<0.001 *	<0.001 *
Vitamin C (mg)	1.0 (0–5.2)	0.3 (0–3.8)	0.5 (0–3.8)	2.6 (0–8.6)	<0.001 *	0.015 *
Vitamin D (µg)	0.1 (0–0.9)	0.1 (0–0.9)	0 (0–0.9)	0 (0–0.9)	0.470	0.895
Vitamin E (mg)	1.6 (0.5–3.3)	1.5 (0.6–3.7)	1.4 (0.5–2.7)	1.8 (0.5–3.4)	0.009 *	0.167
Vitamin A (µg)	156.4 (59.2–356.8)	106.6 (32.3–301.2)	174.2 (61.8–328.2)	211.0 (73.7–447.8)	<0.001 *	0.002 *
Potassium (mg)	244.6 (93.3–467.6)	163.8 (70.4–340.9)	253.9 (88.2–470.4)	316.1 (140.3–696.7)	<0.001 *	0.001 *
Calcium (mg)	120.2 (65.0–222.0)	89.8 (60.6–176.4)	111.0 (59.9–204.0)	174.0 (85.2–322.1)	<0.001 *	<0.001 *
Phosphorus (mg)	195.6 (108.9–303.5)	171.9 (92.5–260.9)	189.5 (107.1–285.4)	240.0 (138.7–373.5)	<0.001 *	<0.001 *
Iron (mg)	2.7 (1.7–5.0)	2.5 (1.7–3.9)	2.5 (1.6–4.6)	3.6 (2.0–6.5)	<0.001 *	<0.001 *
Zinc (mg)	1.3 (0.7–2.7)	1.1 (0.6–2.0)	1.4 (0.6–2.6)	1.6 (1.0–3.0)	<0.001 *	0.187
Magnesium (mg)	30.0 (15.3–59.2)	24.8 (12.1–44.0)	29.6 (14.9–49.8)	52.5 (18.8–75.3)	<0.001 *	<0.001 *
Sodium (mg)	285.7 (114.2–593.1)	359.6 (142.4–764.2)	335.3 (130.5–589.2)	221.1 (78.6–426.2)	<0.001 *	<0.001 *

^1^ SFA: saturated fatty acid; ^2^ *p*-value adjusted for age, sex, urbanity, marital status and level of education (ANCOVA). *statistically significant different at *p* < 0.05 level.

**Table 3:** Breakfast food group intakes across NRF tertiles.

	Total (n = 1263)		NRF Tertile 1 (n = 407)		NRF Tertile 2 (n = 440)		NRF Tertile 3 (n = 416)		*p*-Value ^1^
	Consumers	Median (25th–75th)	Consumers	Median (25th–75th)	Consumers	Median (25th–75th)	Consumers	Median (25th–75th)	
	N (%)		N (%)		N (%)		N (%)		
Cereal grains and pasta (g)	947 (74.9)	120 (100-200)	303 (74.3)	120 (90-200)	321 (73)	120 (90-200)	323 (77.7)	105 (100-180)	0.819
Vegetables and vegetable products (g)	436 (34.5)	45 (30–80)	102 (25.1)	40 (30–60)	138 (31.4)	45 (30–60)	196 (47.1)	60 (30–90)	0.023 *
Legumes and legume products (g)	420 (33.3)	80 (45–100)	80 (19.6)	50 (30–80)	140 (31.8)	75 (40–100)	200 (48.1)	90 (50–125)	<0.001 *
Beverages and sugar-sweetened beverages (g)	233 (18.4)	15 (2–25)	69 (16.9)	14 (7–25)	92 (20.8)	18 (2–25)	72 (17.3)	15 (2–25)	0.968
Finfish and shell fish products (g)	225 (17.8)	40 (30–60)	43 (10.5)	50 (40–70)	74 (16.8)	50 (40–70)	109 (26.2)	40 (20–60)	0.040 *
Snack (g)	219 (17.3)	60 (25–100)	82 (20.1)	50 (25–110)	77 (17.4)	60 (25–100)	60 (14.5)	100 (50–120)	0.286
Eggs and egg products (g)	182 (14.4)	60 (60–60)	78 (19.1)	60 (60–60)	59 (13.4)	60 (60–60)	45 (10.9)	60 (40–60)	0.001 *
Sweets (g)	126 (10)	20 (15–20)	34 (8.4)	20 (15–20)	55 (12.6)	20 (10–20)	36 (8.8)	20 (10–20)	0.538
Poultry products (g)	123 (9.8)	40 (30–60)	35 (8.5)	40 (30–50)	46 (10.5)	50 (30–60)	43 (10.2)	30 (30–50)	0.044 *
Baked products (g)	118 (9.3)	60 (40–75)	53 (12.9)	70 (30–75)	43 (9.7)	54 (40–75)	23 (5.4)	70 (39–75)	0.696
Spices and herbs (g)	63 (5)	10 (10–10)	20 (5)	10 (10–10)	17 (3.8)	10 (5–20)	26 (6.3)	10 (5–10)	0.283
Fats and oils (g)	54 (4.3)	5 (3–10)	27 (6.5)	4 (2–12.5)	20 (4.4)	8 (5–8)	8 (1.9)	5 (2–5)	0.090
Beef products (g)	43 (3.4)	50 (40–90)	17 (4.1)	90 (50–100)	18 (4.2)	40 (30–50)	8 (1.8)	50 (50–50)	0.006 *
Fruits and fruit juice (g)	35 (2.8)	120 (50–200)	11 (2.7)	50 (30–300)	9 (1.9)	120 (100–150)	15 (3.7)	200 (50–200)	0.570
Dairy and dairy products (g)	27 (2.1)	27 (21–40)	5 (1.2)	27 (27–27)	10 (2.3)	40 (21–40)	12 (2.8)	27 (10–200)	0.604

^1^ Kruskal–Wallis test; *statistically significant different at *p* < 0.05 level.

## Data Availability

Public availability of the dataset from the 2018 Indonesian Food Barometer by SEAMEO RECFON based on Open Science philosophy is in progress. Raw data sets are available on request from the corresponding author.

## Supplementary Materials

The following supporting information can be downloaded at https://www.mdpi.com/article/10.3390/nu15173792/s1, Table S1: Sociodemographic Characteristics of Subjects and their association with breakfast habits; Table S2: Median NRF scores among Breakfast Consumers by Sociodemographics.
