# Supplementary material for: Breakfast Consumption and Quality of Macro- and Micronutrient Intake in Indonesia: A Study from the Indonesian Food Barometer
Source: Nutrients. 2023 Aug 30;15(17):3792. doi: 10.3390/nu15173792 (PMC10489646; doi:10.3390/nu15173792)
Supplement: Supplementary file 1 [file nutrients-15-03792-s001.zip › nutrients-2537967-supplementary.pdf]

Supplementary Materials for Manuscript:
Breakfast Consumption and Quality of Macro- and Micronutrient intake in Indonesia: A study from the Indonesian Food Barometer

Table S1. Sociodemographic Characteristics of Subjects and their~~Its~~ Association with Breakfast Habits

		Total	
	Sample N (%)[a]	Skippers N (%)[b]	Consumers N (%)[b]
**All**	1333 (100%)	69 (5.2)	1263 (94.8)
**Urbanity**			
Urban	875 (65.6)	57 (6.5)	818 (93.5)
Rural	458 (34.4)	12 (2.6)	446 (97.4)
p- value[c]~~Valuec~~		*0.002**	
**Age**			
18–~~-~~29 years	399 (29.9)	29 (7.3)	270 (92.7)
30–~~-~~59 years	769 (57.7)	30 (3.9)	739 (96.1)
≥60 years	166 (12.5)	11 (6.6)	155 (93.4)
p-v~~V~~alue[c]		*0.035**	
**Sex**			
Male	692 (51.9)	38 (5.4)	654 (94.6)
Female	641 (48.1)	32 (4.9)	609 (95.1)
p-v~~V~~alue[c]		*0.683*	
**Religion**			
Mu~~o~~sli~~e~~m	1235 (92.7)	61 (4.9)	1174 (95.1)
Non-Mu~~o~~sli~~e~~m	97 (7.3)	8 (8.4)	89 (91.6)
p-v~~V~~alue[c]		*0.157*	
**Marital status**			
Single/widow/divorce	409 (30.7)	36 (8.9)	372 (91.1)
Married/living together	924 (69.3)	33 (3.5)	891 (96.5)
p-v~~V~~alue[c]		*<0.001**	
**Education**			
≤ Junior high school	601 (45.1)	28 (4.7)	573 (95.3)
Senior high school	577 (43.2)	36 (6.2)	541 (93.8)
University degree	153 (11.5)	5 (3.1)	148 (96.9)
p-v~~V~~alue[c]		*0.248*	
**Wealth index**			
T1	451 (33.8)	26 (5.8)	425 (94.2)
T2	437 (32.8)	23 (5.4)	413 (94.6)
T3	444 (33.3)	19 (4.4)	425 (95.6)
p-v~~V~~alue[c]		*0.590*	
**Nutritional status**			
Underweight	132 (9.9)	13 (10)	118 (90)
Normal	723 (54.2)	38 (5.2)	685 (94.8)
Overweight	240 (18.0)	7 (3)	233 (97)
Obese	236 (17.7)	11 (4.7)	225 (95.3)
p-v~~V~~alue[c]		*0.035**	

[a]colomn %, [b]row %, [c]*Chi-Square Test; p <0.05 considered significant*

Formatted: Font: Italic, Complex Script Font: Italic

Formatted: Font: Not Italic, Complex Script Font: Not Italic

Formatted: Font: Not Italic, Complex Script Font: Not Italic

Supplementary Table S2. Median NRF 9.3 Scores among Breakfast Consumers by Sociodemographics

	NRF Score of Breakfast Consumers	
	**N**	**Median (25th–-75th)**
**All**	1332	383.5 (295.2–-471.0)
**Breakfast Consumption**		
Skipper	69	401.7 (321.3–-453.9)
Consumer	1263	383.4 (295.2–-471.1)
p-vValue		*0.605*
	Among Breakfast consumers	
**Age**		
18–-29 years	370	341.5 (260.3–-432.7)
30–-59 years	739	393.2 (311.2–-478.9)
≥60 years	155	414.9 (325.9–-484.4)
p-vValue		*<0.001**
**Sex**		
Male	654	392.1 (298.2–-475.3)
Female	610	371.9 (287.8–-463.5)
p-vValue		*0.033**
**Urbanity**		
Urban	818	368.8 (276.5–-454.8)
Rural	446	411.4 (332.6–-484.0)
p-vValue		*<0.001**
**Religion**		
Muoslieem	1174	380.3 (289.4–-471.0)
Non-Muoslieem	89	391.6 (332.3–-470.5)
p-vValue		*0.209*
**Marital status**		
Single/widow/divorce	372	367.9 (274.4–-463.6)
Married/living together	891	391.6 (298.4–-472.7)
p-vValue		*0.011**
**Education**		
≤ Junior high school	573	408.5 (312.8–-481.0)
Senior high school	541	363.9 (275.5–-439.3)
University degree	148	377.1 (279.2–-524.5)
p-vValue		*<0.001**
**Wealth index**		
T1	425	396.0 (311.2–-483.5)
T2	413	379.5 (283.3–-451.8)
T3	425	371.9 (282.5–-474.6)
p-vValue		*0.002**
**Nutritional status**		
Underweight	118	362.6 (246.4–-444.6)
Normal	685	379.0 (297.2–-475.4)
Overweight	233	384.0 (311.3–-458.5)
Obese	225	403.8 (294.2–-476.7)
p-vValue		*0.027**

Formatted: Font: Italic, Complex Script Font: Italic
